# Both symbionts and environmental factors contribute to shape the microbiota in a pest insect, *Sogatella furcifera*

**DOI:** 10.3389/fmicb.2023.1336345

**Published:** 2024-01-24

**Authors:** Kun Yang, Hua-Yue Zhang, Peng Wang, Gui-Xiu Jin, Dong Chu

**Affiliations:** ^1^Shandong Engineering Research Center for Environment-friendly Agricultural Pest Management, College of Plant Health and Medicine, Qingdao Agricultural University, Qingdao, China; ^2^Shandong Province Centre for Bioinvasions and Eco-security, Qingdao, China; ^3^Linyi Academy of Agricultural Sciences, Linyi, China

**Keywords:** *Sogatella furcifera*, *Wolbachia*, *Cardinium*, 2bRAD-M sequencing, environmental factors

## Abstract

**Introduction:**

Bacterial symbionts are prevalent in arthropods globally and play a vital role in the fitness and resistance of hosts. While several symbiont infections have been identified in the white-backed planthopper Sogatella furcifera, the impact of environmental factors on the microbiota within *S. furcifera* remains elusive.

**Methods:**

In this study, a total of 142 S. furcifera individuals from 18 populations were collected from 14 locations across six countries (China, Thailand, Myanmar, Cambodia, Vietnam, and Laos) analyzed with 2bRAD-M sequencing, to examine the effects of symbionts on the microbiota in the *S. furcifera* population, as well as the vital effects of environmental factors on the bacterial communities.

**Results and discussion:**

Based on the results, in *S. furcifera*, the presence of symbionts Wolbachia and Cardinium negatively influenced the abundance of other bacteria, including Enterobacter, Acinetobacter, and Lysinibacillus, while Wolbachia infection significantly decreased the diversity of the microbial community. Moreover, several environmental factors, including longitude, latitude, temperature, and precipitation, affected the abundance of symbionts and microbiota diversity in *S. furcifera*. These results collectively highlight the vital role of Wolbachia in *S. furcifera* microbiota, as well as the intricate effects of environmental factors on the bacterial communities of *S. furcifera*.

## 1 Introduction

As a significant pest in China and South Asia, the white-backed planthopper (WBPH), scientifically referred to as *Sogatella furcifera* (Hemiptera: Delphacidae), causes substantial losses on agricultural fields, particularly rice paddies (Savary et al., 2012; Huang and Qin, 2018). *S. furcifera* causes damage by sucking phloem sap from rice plants and transmitting viruses, including the southern rice black-streaked dwarf virus, to rice hosts (Zhou et al., 2008, 2018). Over the past decade, the frequency of *S. furcifera* outbreaks has dramatically increased, making it a destructive pest in rice production (Hu et al., 2015; Wang et al., 2018). With its high adaptability, long-distance migratory abilities, and the development of resistance to existing pesticides, managing *S. furcifera* outbreaks has become increasingly challenging (Wang et al., 2019). Coupled with the growing problem of pesticide residue, there is an urgent need to develop novel and efficient methods to mitigate the damage elicited by pest insects, including *S. furcifera*.

Microbial communities, especially bacteria, including endosymbionts, play a vital role in the development, fitness, fecundity, and resistance of host arthropods (Douglas, 2009; McFall-Ngai et al., 2013). These symbiotic bacteria fall into two categories: primary symbionts (obligate symbionts) and secondary symbionts (facultative symbionts), residing predominantly in bacteriocytes within hosts and exerting complex influences on host arthropods (Werren et al., 2008; Zeng et al., 2018; Yang et al., 2022). The former is essential for the development and survival of host invertebrates. For instance, the symbiont *Portiera* provides essential amino acids (EAA) and B vitamins to whitefly hosts (Wang et al., 2022), whilst the symbiont *Buchnera* supplies aphid hosts with various nutritional elements and protects them from heat stress (Dunbar et al., 2007; Douglas, 2009).

*Wolbachia*, the most renowned symbiont in invertebrates, was first discovered in mosquitoes in 1924 (Hertig and Wolbach, 1924). Interestingly, it can infect at least 66% of all arthropod species (Werren, 1997; Miao et al., 2020). In filarial nematodes, *Wolbachia* is regarded as an obligate mutualist, essential for the development and fertility of host nematodes (Taylor et al., 2005; Wasala et al., 2019). Previously considered reproductive parasitism in invertebrates, capable of manipulating host reproduction through phenomena such as cytoplasmic incompatibility, feminization, parthenogenesis, and male-killing (Werren et al., 2008), recent studies have established the benefits conferred by *Wolbachia* (Zug and Hammerstein, 2015). Another study corroborated the influence of *Wolbachia* on microbial communities in small planthoppers (Duan et al., 2020). However, the intricate interaction between *Wolbachia* and the bacterial community in arthropods warrants further investigations.

Microbial communities, including symbionts in arthropods, are influenced by various biotic and abiotic factors. For example, high temperatures significantly influence symbiont titer and the diversity of the bacterial community in whiteflies (Yang et al., 2022). Similarly, the diet of invertebrate hosts significantly impacts the structure of microbial communities (Santo Domingo et al., 1998; Colman et al., 2012), and both temperature and humidity affect bacterial communities (Behar et al., 2008). Furthermore, earlier research has documented the crucial impact of host genetic backgrounds on microbiota (Suzuki et al., 2019; Gupta and Nair, 2020). Interestingly, the presence of symbionts exerts a significant influence on microbial communities. Symbionts occupy a relatively large space and engage in competition with other bacteria for nutrients. According to a prior study, *Spiroplasma* infection significantly decreases the titer of *Wolbachia* in the same host (Goto et al., 2006). Additionally, endosymbionts such as *Wolbachia* and *Cardinium* can lower the microbiome diversity of the host *Sogatella furcifera* (Li et al., 2022). The complex effects of symbionts on the microbiota of *S. furcifera* require further investigation.

2bRAD-M sequencing is a novel method that is efficient in detecting low-biomass microbiomes at the species level with high fidelity (Sun et al., 2022). In recent years, the damage caused by *S. furcifera* has drammatically rapidly, particularly in Asia and China. However, the complex interactions between the bacterial community of *S. furcifera,* including bacterial symbionts and environmental factors, remain poorly understood. Moreover, the potential application of symbionts in the biocontrol of *S. furcifera* is an area that warrants exploration. In this study, 18 *S. furcifera* populations sourced from six Asian countries were subjected to 2bRAD-M sequencing in order to unravel the vital factors contributing to shaping microbial communities in *S. furcifera*. Moreover, this comprehensive study explored the effects of symbionts and environmental factors on bacterial communities.

## 2 Materials and methods

### 2.1 Collection of *Sogatella furcifera* populations in China and South Asia

To determine the relationships among symbionts, bacterial communities in *S. furcifera,* and environmental factors, a total of 142 *S. furcifera* individuals from 18 populations were collected from 14 locations across six countries (China, Thailand, Myanmar, Cambodia, Vietnam, and Laos) analyzed in this study. Each *S. furcifera* population comprised at least six independent samples. All *S. furcifera* samples were preserved in 100% alcohol and subsequently dispatched for 2bRAD-M sequencing. Information about the samples is detailed in Table 1 and Supplementary Table S1, wherein each replicate represents an independent *S. furcifera* adult.

**Table 1:** Collection information of *Sogatella furcifera* populations used in this experiment.

Population name	Collection location	Longitude	Latitude	Elevation	Collection date	Replicates	Host plants
VE	Vientiane, Laos	N18.215171	E102.502263		2014.3.18	12	Rice
SA	Sawakeexay Village,Hinhoun District,Khammvean Province. Laos	N17.724459	E104.567768	130	2014.3.20	8	Rice
1 M	Rangoon, Myanmar	N16.78333	E96.16667	15		8	Rice
2 M	Rangoon, Myanmar	N16.78333	E96.16667	15		8	Rice
CH	Changmai-Mangkok, Thailand	N16.487457	E99.485583	53	2014.5.14	7	Rice
RO	Royal university of agriculture, Cambodia	N11.513352	E104.901175	12	2014.3.24	6	Rice
KO	KomReurk,district,Siem Reap city,Siew Reap province, Cambodia	N13.336719	E103.661119	7	2014.3.27	6	Rice
LU	Luong Ninh commune,Quang Ninh district,Quang Ninh province, Vietnam	N17.428303	E106.633235	10	2014.4.17	6	Rice
LO	Los son commune,Phu loo district,Hue province, Vietnam	N16.331932	E107.750584	1.4	2014.4.18	8	Rice
BS	Baoshan, Yunnan Province, China	N25.057464	E99.163658	1699.9	2014.6.26	8	Rice
CX	Chuxiong, Yunnan Province, China	N25.086440	E101.467300	1812.8	2014.6.26	8	Rice
FN	Funing, Yunnan Province, China	N23.625847	E105.630900	680	2014.6.10	8	Rice
GM	Gengma, Yunnan Province, China	N23.538747	E99.397175	1,116	2014.7.10	8	Rice
JY	Jiyang, Jinan, Shandong Province, China	N36.972935	E117.21142	21		8	Rice
MH	Menghai, Yunnan Province, China	N21.966323	E100.449572	1,230	2014.5.15	8	Rice
TC	Tancheng, Linyi, Shandong Province, China	N34.61354	E118.36712	47		6	Rice
YT	Yutai, Jining, Shandong Province, China	N35	E116.65	35		7	Rice
ZY	Zhaoyang, Yunnan Province, China	N27.320429	E103.706542	1907	2014.7.10	12	Rice

One replicate in Table 1. Means an independent female adult sequenced by 2bRAD-M in this experiment.

### 2.2 DNA extraction and 2bRAD-M sequencing

Bacterial communities in *S. furcifera* were analyzed using the 2bRAD-M sequencing methods. 2bRAD-M sequencing and library construction were performed by Qingdao OE Biotech Co., Ltd. (Qingdao, China), following previously established protocols (Wang et al., 2012) with marginal modifications. Briefly, the DNA (100 ng) from each sample was digested using 4 U of the BcgI enzyme (NEB) at 37°C for 3 h. Next, the resulting DNA fragments were ligated with specific adaptors. A mix of 5 μL of digested DNA and 10 μL of ligation master mix, containing 0.2 μM of each adaptor and 800 U of T4 DNA ligase (NEB), underwent a ligation reaction at 4°C for 12 h.

Following ligation, the products were PCR amplified, and the resulting DNA was subjected to electrophoresis on an 8% polyacrylamide gel. An approximately 100-bp band was excised, and the DNA fragments were then diffused in DEPC water at 4°C for 12 h. A PCR test was conducted using primers bearing platform-specific barcodes to introduce sample-specific barcodes. Each PCR sample, totaling 20 μL, contained 25-ng of gel-extracted PCR product, 0.2 μM each of forward and reverse primers, 0.3 mM dNTP mix, 0.4 U Phusion high-fidelity DNA polymerase (NEB), and 1 × Phusion HF buffer. After PCR amplification and electrophoresis, the PCR products were purified using the QIAquick PCR purification kit (50) (Qiagen) and sequenced on the Illumina Nova PE150 platform.

### 2.3 Data analysis of 2bRAD-M sequencing results

To conduct the 2bRAD-M analysis, microbial genome data from the NCBI database, consisting of 173,165 species of fungi, bacteria, and archaea, were employed. Restriction enzymes of 16 type 2B were utilized to fragment the samples, and Perl scripts were used in this process. Thereafter, the RefSeq (GCF) number was used to assign 2bRAD-M tags with microbial genome information, including taxonomic data. Unique 2bRAD tags occurring only once in every GCF were selected as species-specific 2bRAD-M markers, forming a reference database. A detection threshold of 0.0001 (0.01%) relative abundance was set as the default (Franzosa et al., 2018).

To calculate the relative abundance of each bacterium, 2bRAD tags from all samples, post-quality control, were mapped against the 2bRAD marker database containing tags unique to the 26,163 microbial species using a built-in Perl script. To mitigate false positives in species identification, a G score was calculated for each identified species within a given sample. This score, derived from a harmonic means of read coverage of 2bRAD markers belonging to a species and the total number of possible 2bRAD markers for this species, was employed identify species while minimizing errors. A threshold G score of 5 was set to prevent false-positive microbial species discovery (Sun et al., 2022).


$$G\;{\mathrm{score}}_{\mathrm{species}\;i}=\sqrt{S_{i}\times t_{i}}$$

**S:** the number of reads assigned to all 2bRAD markers belonging to species *i* within a sample.

**T:** number of all 2bRAD markers of species i that have been sequenced within a sample.

The average read coverage of all 2bRAD markers for each species was computed to represent the number of individuals of a species present within a sample at a given sequencing depth. The relative abundance of a species was then calculated as the ratio of the number of microbial individuals belonging to that species against the total number of individuals from known species detected within a sample.


Relativeabundancespeciesi=SiTi∑i=1nSiTi

**S**: the number of reads assigned to all 2bRAD markers of species *i* within a sample.

**T**: the number of all theoretical 2bRAD markers of species *i*.

**Figure 1:**
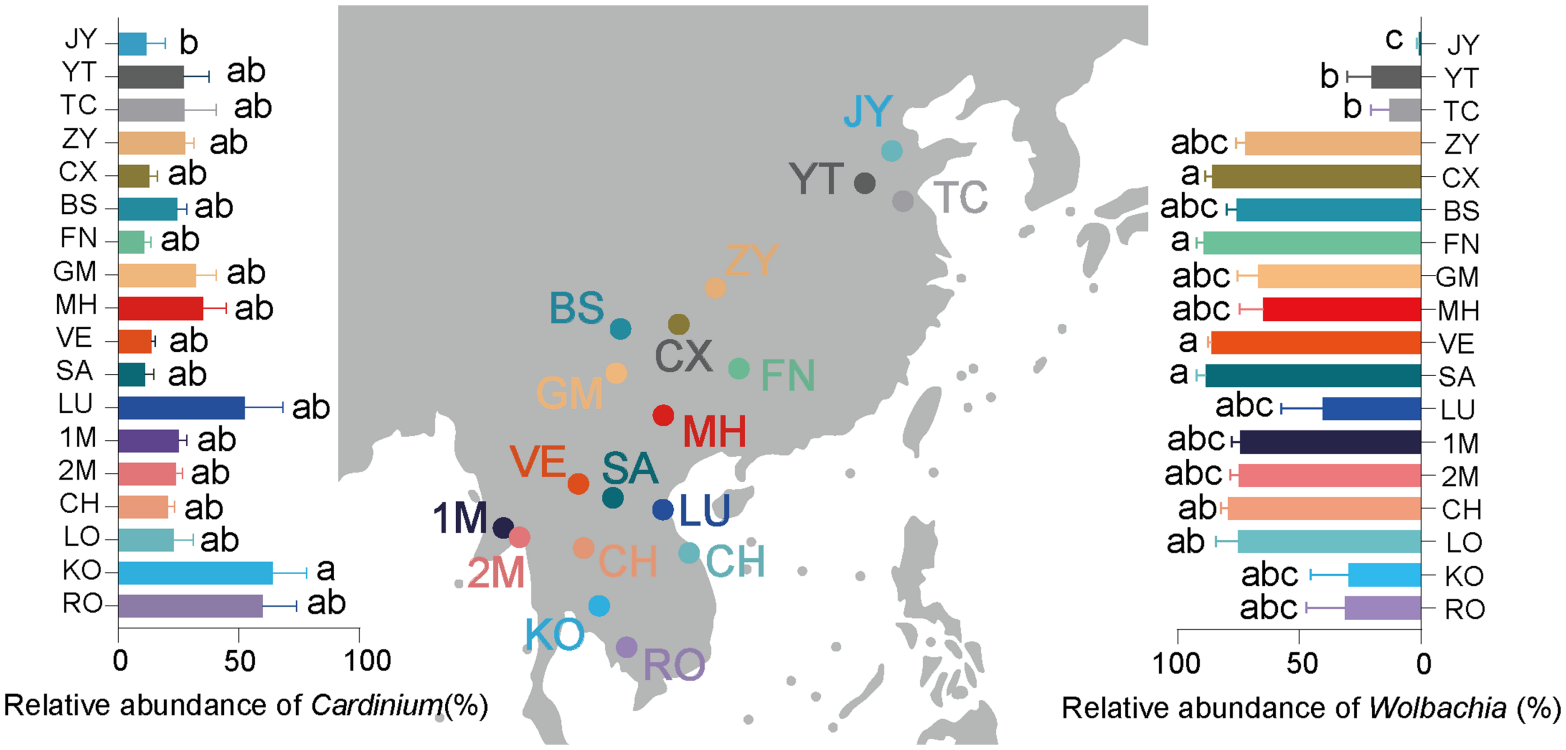
Relative infection abundance of *Cardinium* (left panel), *Wolbachia* (right panel) and collection sites (middle panel) of *Sogatella furcifera* across 18 geographical locations by 2bRAD-M sequencing. As the symbiont infection data do not follow a normal distribution, the difference of *Wolbachia* abundance in various *S. furcifera* populations was analyzed by Kruskal–Wallis test and Dunn’s test with Bonferroni correction for multiple comparisons by SPSS 21.0, different letters represent significant difference.

**Figure 2:**
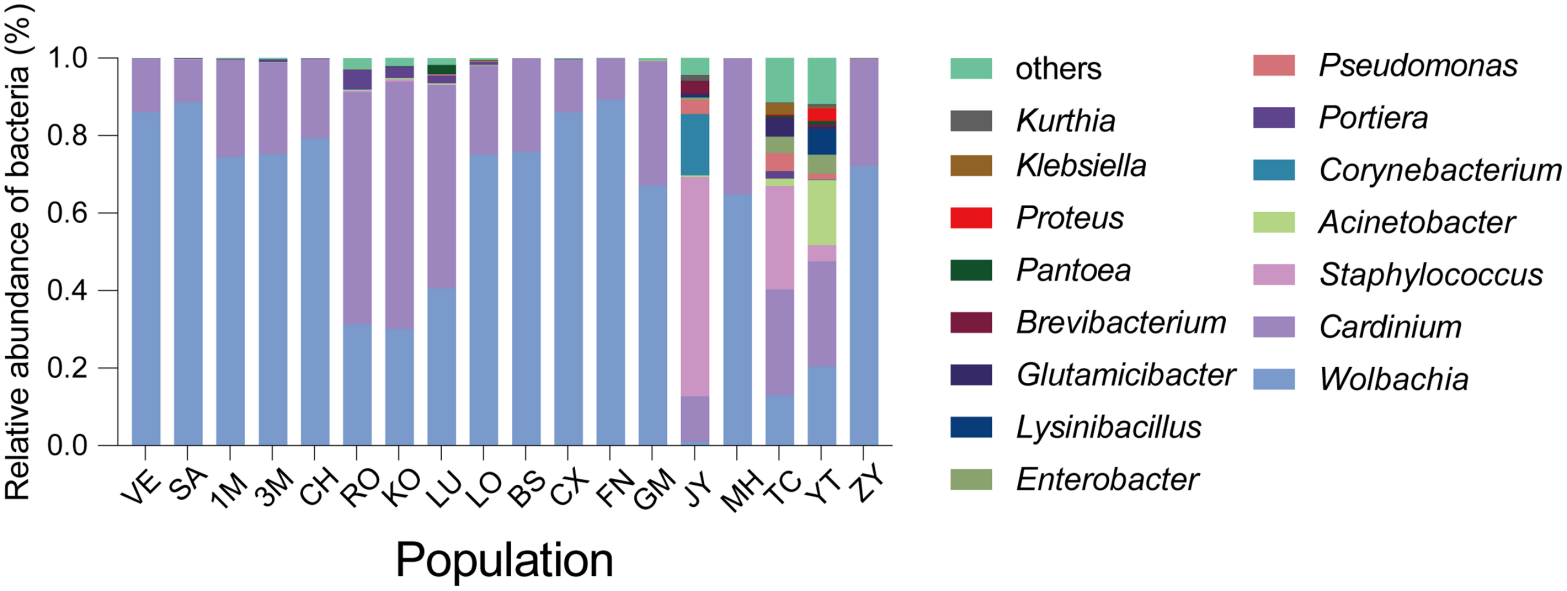
The abundance of microbial communities of *Sogatella furcifera* across 18 populations collected in Asia based on 2bRAD-M sequencing results. The relative abundance of top 15 abundant bacteria at the genus level was shown with different populations.

Additionally, five environmental factors, namely annual mean temperature, annual precipitation, longitude, latitude, and altitude, were analyzed in the study. Data for these factors were downloaded from the WorldClim website.^1^ The impacts of climate and geographical factors on diversity and abundance indexes and symbiont infections were described using two structural equation models (SEM) with Satorra-Bentler correction. To limit heteroscedasticity, the log value of the “precipitation” values was used in the SEMs. SEM models were deemed acceptable when *p* value >0.05 and CFI > 0.95, after systematically excluding redundant pathways based on a lower AIC value. To standardize each parameter and eliminate variance, SEM coefficients were estimated through standardized transformation. Prior to analysis, data on *Cardinium* abundance (shown in Figure 1 left panel), *Wolbachia* abundance (shown in Figure 1 right panel), and microbial diversities of samples (shown in Figure 2) underwent normality testing using the Kolmogorov–Smirnov test and Levene’s test to assess the homogeneity of group variances. Data exhibiting a normal distribution were analyzed using one-way ANOVA with post-hoc Tukey HSD. In cases where the *Cardinium* abundance, *Wolbachia* abundance, and diversity data did not conform to normal distribution, they were analyzed using the Kruskal-Wallis test and Dunn’s test with Bonferroni correction for multiple comparisons. All statistical analyzes were conducted using SPSS 21.0. PCoA plots illustrating Bray-Curtis intersample distances and classification probabilities were generated using QIIME software (Caporaso et al., 2010). Pearson correlation analysis, performed using SPSS 21.0, was used to explore the relationships between different symbionts and diversity indexes. Furthermore, all graphical representations were generated using GraphPad Prism 9.0.0.

## 3 Results

### 3.1 Composition of bacterial communities in different *Sogatella furcifera* populations

**Figure 3:**
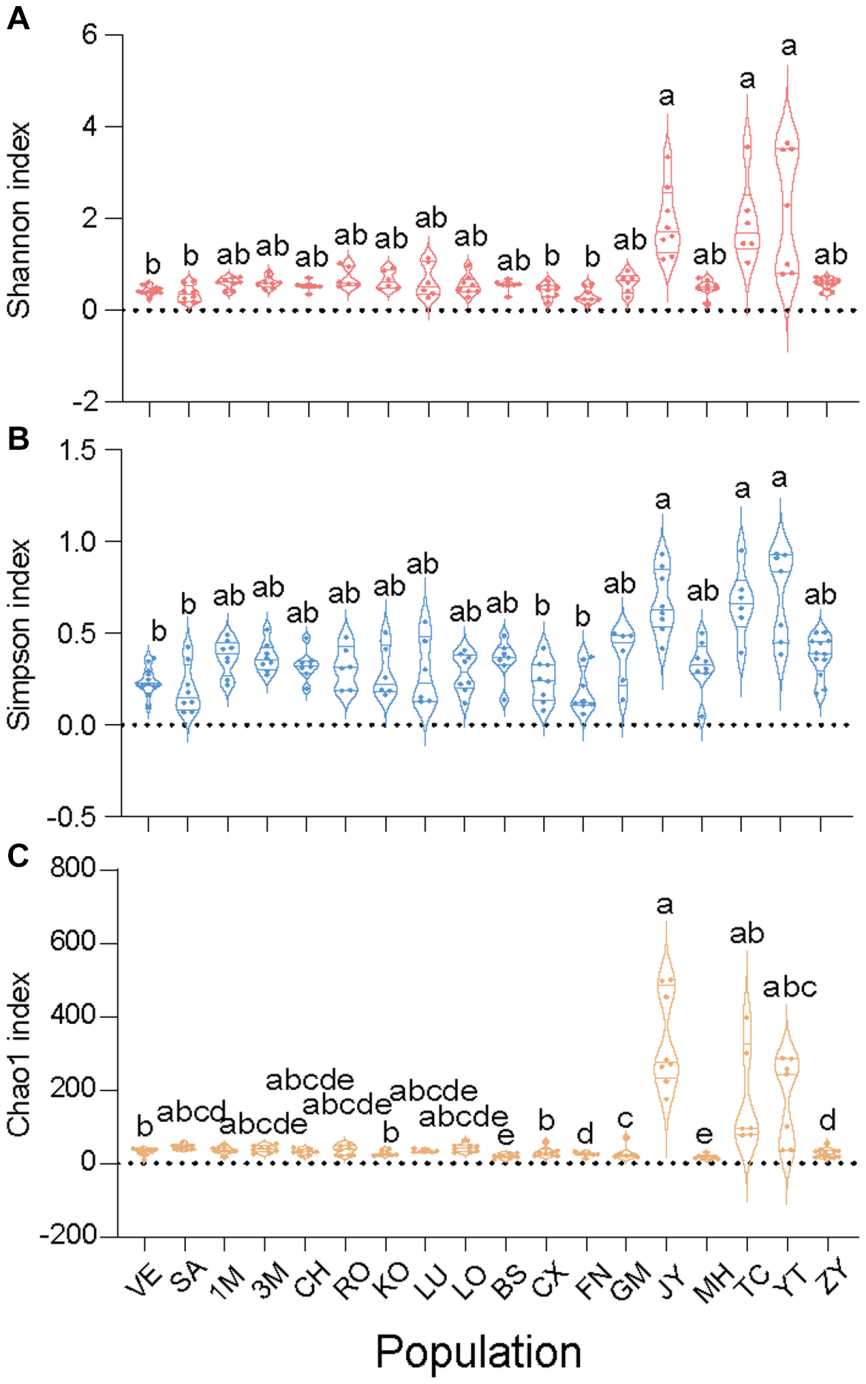
Alpha diversities of all 18 *Sogatella furcifera* MED populations collected in Asia. The Shannon index **(A)**, Simpson index **(B)** and Chao1 index **(C)** were present based on 2bRAD-M sequencing results, respectively. As the diversity data do not follow a normal distribution, they were analyzed by Kruskal–Wallis test and Dunn’s test with Bonferroni correction for multiple comparisons by SPSS 21.0, different letters represent significant difference.

In the current study, a total of 18 *S. furcifera* populations were analyzed, comprising 9 populations from China and 9 populations from the South Asian region. The predominant bacterial symbionts identified in all *S. furcifera* populations were *Wolbachia* and *Cardinium*. Interestingly, the former was the most abundant bacteria in nearly all *S. furcifera* populations, being present in every *S. furcifera* sample. Notably, the relative abundance of *Wolbachia* exceeded 80% in two Chinese populations, specifically CS (85.95 ± 2.92%, Mean ± SEM) and FN (89.26 ± 3.01%, Mean ± SEM), as well as in all Laos populations, including VE (86.02 ± 1.57%, Mean ± SEM) and SA (86.02 ± 3.58%, Mean ± SEM). Nevertheless, significant variations in *Wolbachia* abundance were observed among different populations. Meanwhile, 3 *S. furcifera* populations from China (JY, YT, and TC) exhibited the lowest *Wolbachia* abundance, significantly lower than the *Wolbachia*-abundant populations such as FN, VE, and SA (Figure 1 right panel). *Cardinium* emerged as the second most abundant bacterium in *S. furcifera* (Figure 1 left panel). In several South Asia populations, including KO (63.88 ± 14.38%, Mean ± SEM), RO (59.96 ± 14.04%, Mean ± SEM), and LU (52.50 ± 15.74%, Mean ± SEM) as well as one Chinese population, YT (27.18 ± 10.38%, Mean ± SEM) *Cardinium* was the dominant symbiont, with its abundance in the KO population being significantly higher than that in the JY population (11.72 ± 7.78%, Mean ± SEM) (*p* < 0.05, Kruskal-Wallis test). Besides, *Wolbachia* abundance was less than 1% in the JY population, while *Cardinium* abundance was the highest (56.59 ± 11.90%, Mean ± SEM). Additionally, the primary symbiont *Portiera*, typically associated with whiteflies, was also detected in several *S. furcifera* populations, including TC, KO, VE, etc. (Figure 3). Principal Component Analysis was conducted to explore differences among bacterial communities of all *S. furcifera* populations, revealing distinct microbial communities in *S. furcifera* individuals from FN and JY populations compared to other samples (Supplementary Figure S1).

### 3.2 Microbial diversities of different geographical *Sogatella furcifera* populations

To elucidate variations in microbial communities among different *S. furcifera* populations, alpha diversity indices, including Shannon, Simpson, and Chao1, were calculated for each *S. furcifera* population. Considering that the three alpha indices of all populations did not follow a normal distribution, the Kruskal–Wallis test, and Dunn’s test with Bonferroni correction for multiple comparisons were adopted (Figure 2). Intriguingly, populations with low *Wolbachia* abundance exhibited higher alpha diversities in bacterial communities compared to those with high *Wolbachia* abundance. Specifically, populations with low *Wolbachia* abundance, such as JY, TC, and YT, displayed significantly higher Shannon and Simpson indices compared to populations with high *Wolbachia* abundance, including VE, SA, CX, and FN (Figure 2). The UPGMA hierarchical cluster diagram of different *Sogatella furcifera* populations with 2bRAD-M sequencing data was shown (Supplementary Figure S2), and the phylogenetic tree of different *Sogatella furcifera* populations with 2bRAD sequencing results was constructed (Supplementary Figure S3).

### 3.3 Influence of *Wolbachia* and *Cardinium* on other bacteria and microbial diversities of *Sogatella furcifera*

**Figure 4:**
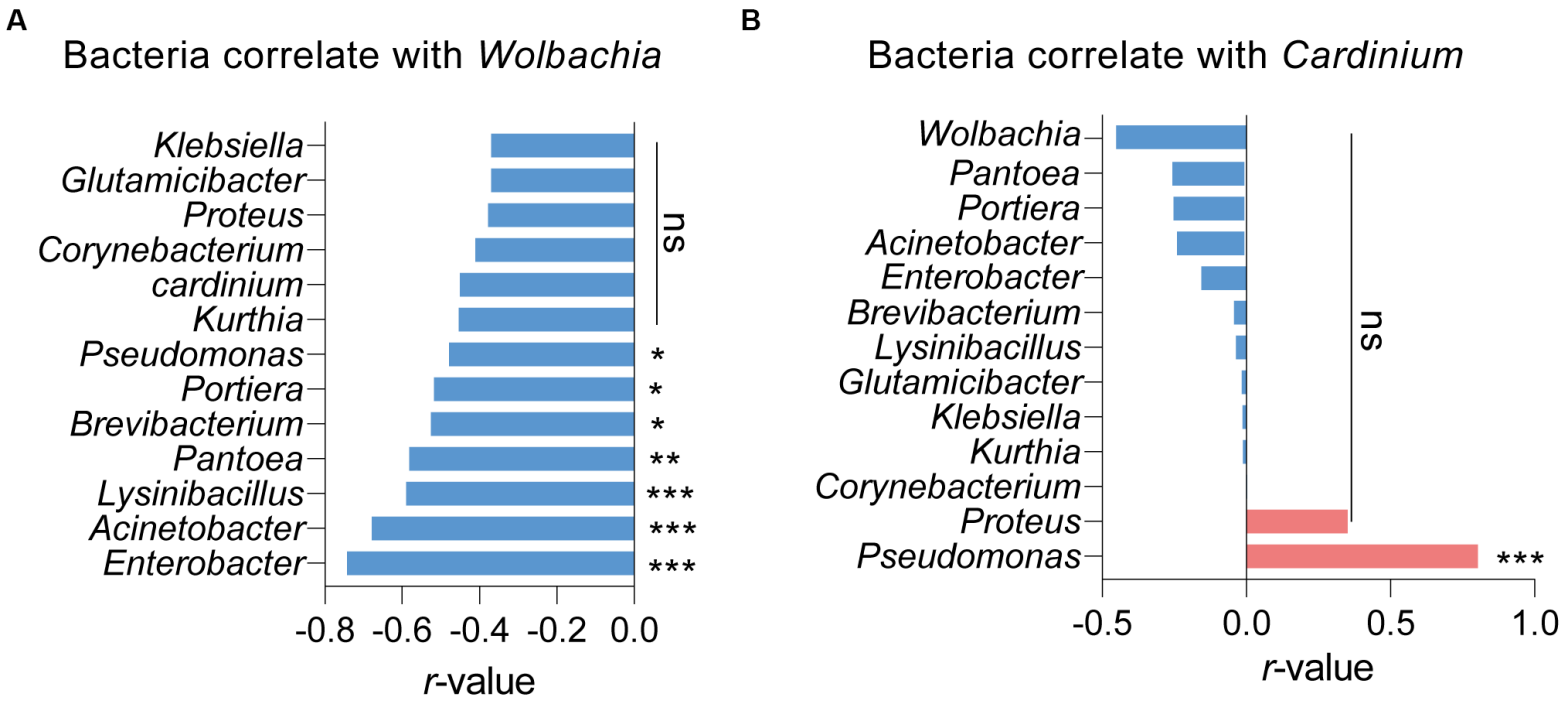
Relationships between the proportions of other main 13 bacteria and the proportions of *Wolbachia* **(A)** or the proportions of *Cardinium* **(B)** among all 18 *Sogatella furcifera* populations by Pearson correlation analysis (SPSS 21.0) based on 2bRAD-M sequencing results. *r*-values and *p*  values of each linear regression plots are provided. “ns” means no significant; asterisks indicate significant difference the two compared group, *p  < 0.05; **p  < 0.01; ***p  < 0.001.

Pearson analysis was used to assess the correlations between symbionts and other bacteria in *S. furcifera*. As illustrated in Figure 4A, the presence of *Wolbachia* negatively impacted the abundance of various bacteria, encompassing *Enterobacter*, *Acinetobacter*, and *Lysinibacillus*. Notably, *Wolbachia* significantly and negatively influenced the abundance of *Portiera* in *S. furcifera* (*r* = −0.518, *p* < 0.05, Pearson analysis). While the abundance of *Wolbachia* was negatively correlated with that of *Cardinium*, the correlation was not statistically significant (*r* = −0.451, *p* = 0.06, Pearson analysis). Consistently, *Cardinium* significantly positively influenced the abundance of *Pseudomonas* (*r* = −0.804, *p* < 0.001, Pearson analysis) and that of various other bacteria, albeit the correlation was not statistically significant (Figure 4B).

**Figure 5:**
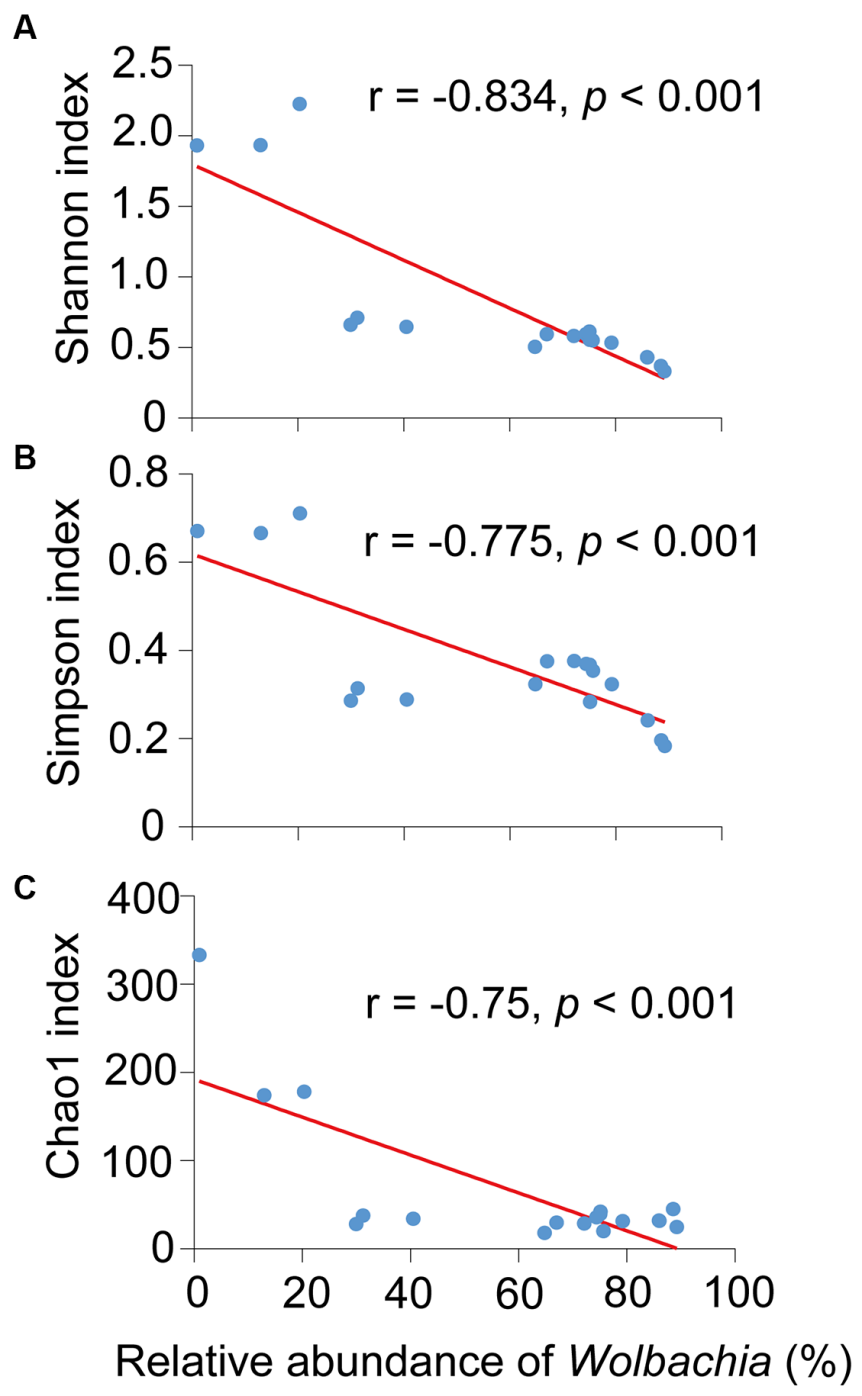
Relationships between the abundance of *Wolbachia* and the Shannon diversity index **(A)**, Simpson index **(B)** and Chao1 index **(C)** among all 18 *Sogatella furcifera* populations by Pearson correlation analysis (SPSS 21.0) based on 2bRAD-M sequencing results. *r*-values and *p* values of each linear regression plots are provided.

Noteworthily, Pearson analysis exposed that the presence of *Wolbachia* significantly and negatively affected the three diversity indexes of *S. furcifera*, namely the Shannon index (*r* = −0.834, *p* < 0.001, Pearson analysis), Simpson index (*r* = −775, *p* < 0.001, Pearson analysis), and Chao1 index (*r* = −0.750, *p* < 0.001, Pearson analysis) (Figure 5). *Cardinium* infection also had a negative impact on the diversity indexes, although the correlations were not significant (Supplementary Figure S4).

### 3.4 Effects of environmental factors on bacterial abundance, including symbionts in *Sogatella furcifera*

**Figure 6:**
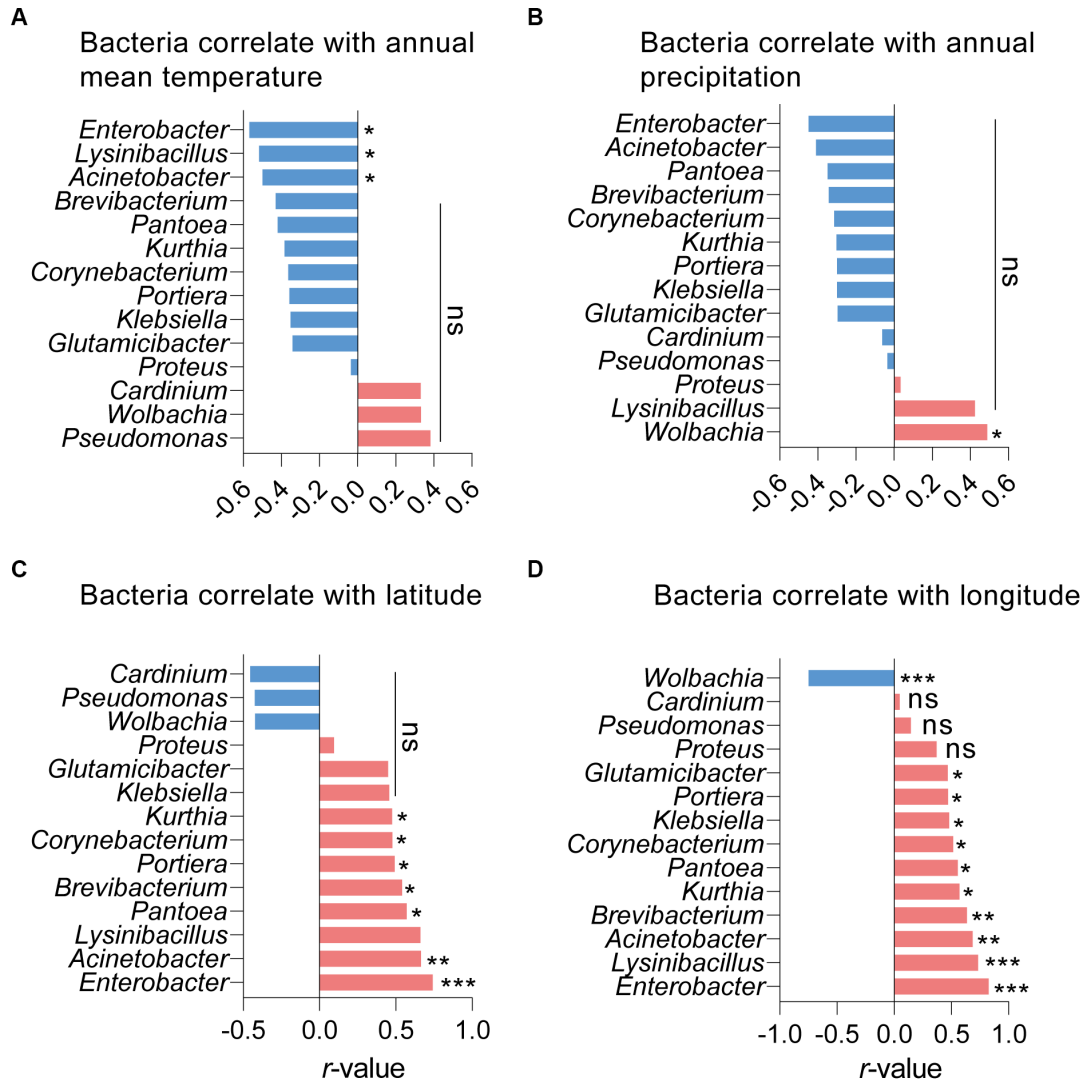
Relationships between the proportions of main 14 bacteria and the annual mean temperature **(A)**, annual precipitation **(B)**, latitude **(C)** and longitude **(D)** among all 18 *Sogatella furcifera* populations by Pearson correlation analysis (SPSS 21.0) based on 2bRAD-M sequencing results. *r*-values and *p*  values of each linear regression plots are provided. “ns” means no significant; asterisks indicate significant difference the two compared group, *p  < 0.05; **p  < 0.01; ***p  < 0.001.

The effects of five environmental factors (annual mean temperature, annual precipitation, latitude, longitude, and altitude) on *S. furcifera* microbiota were determined through Pearson analysis. While both *Wolbachia* and *Cardinium* abundance in *S. furcifera* increased with annual mean temperature, there was no significant correlation between temperature and the abundance of these two symbionts. Notwithstanding, temperature had a significantly negative impact on the abundance of numerous bacteria, such as *Enterobacter*, *Lysinibacillus,* and *Acinetobacter* (Figure 6A). Precipitation had a significant positive influence on *Wolbachia* abundance (*r* = 0.489, *p* < 0.05, Pearson analysis) (Figure 6B). Latitude was significantly and positively correlated with the abundance of many bacteria and was negatively correlated with that of *Cardinium* and *Wolbachia*, although the difference was not significant for the latter (Figure 6C). On the other hand, longitude was significantly and negatively correlated with the abundance of *Wolbachia* (*r* = 0.750, *p* < 0.001, Pearson analysis) and positively correlated with the abundance of other bacteria in *S. furcifera* (Figure 6D). Finally, altitude did not significantly affect bacterial abundance in *S. furcifera* (Supplementary Figure S5).

**Figure 7:**
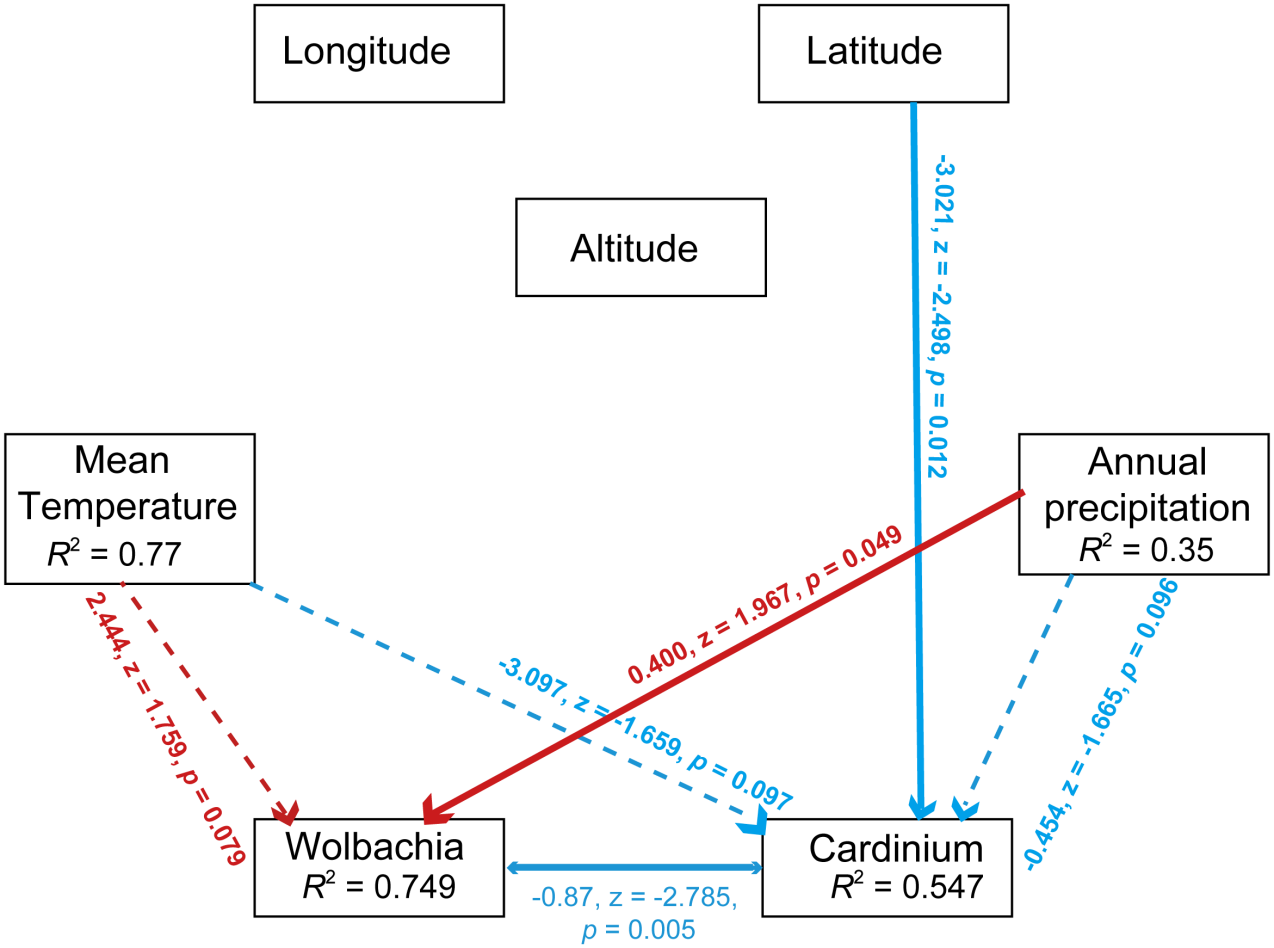
Path diagram for the structural equation model (SEM) for environmental factors (including annual mean temperature, annual precipitation, longitude, altitued and latitude) and symbionts’ abundance (including *Wolbachia* and *Cardinium*). Statistically significant negative paths are indicated by blue arrows, while positive paths are indicated by red arrows. The *r* values in each box indicate the amount of variation in that variable explained by the input arrows. Numbers next to arrows are unstandardized slopes.

SEM analysis validated the significant influence of precipitation on *Wolbachia* abundance (*r* = 0.400, z = 1.967, *p* < 0.05, SEM) and the negative effect of latitude on *Cardinium* abundance (*r* = −3.021, z = −2.498, *p* < 0.05, SEM). In contrast to Pearson analysis, SEM analysis revealed a significant negative correlation between *Wolbachia* and *Cardinium* (*r* = −0.87, z = −2.785, *p* < 0.01, SEM). Lastly, temperature had a numerically positive effect on *Wolbachia* abundance and a negative effect on *Cardinium* abundance (Figure 7).

### 3.5 Influence of environmental factors on microbial diversities in *S. furcifera*

**Figure 8:**
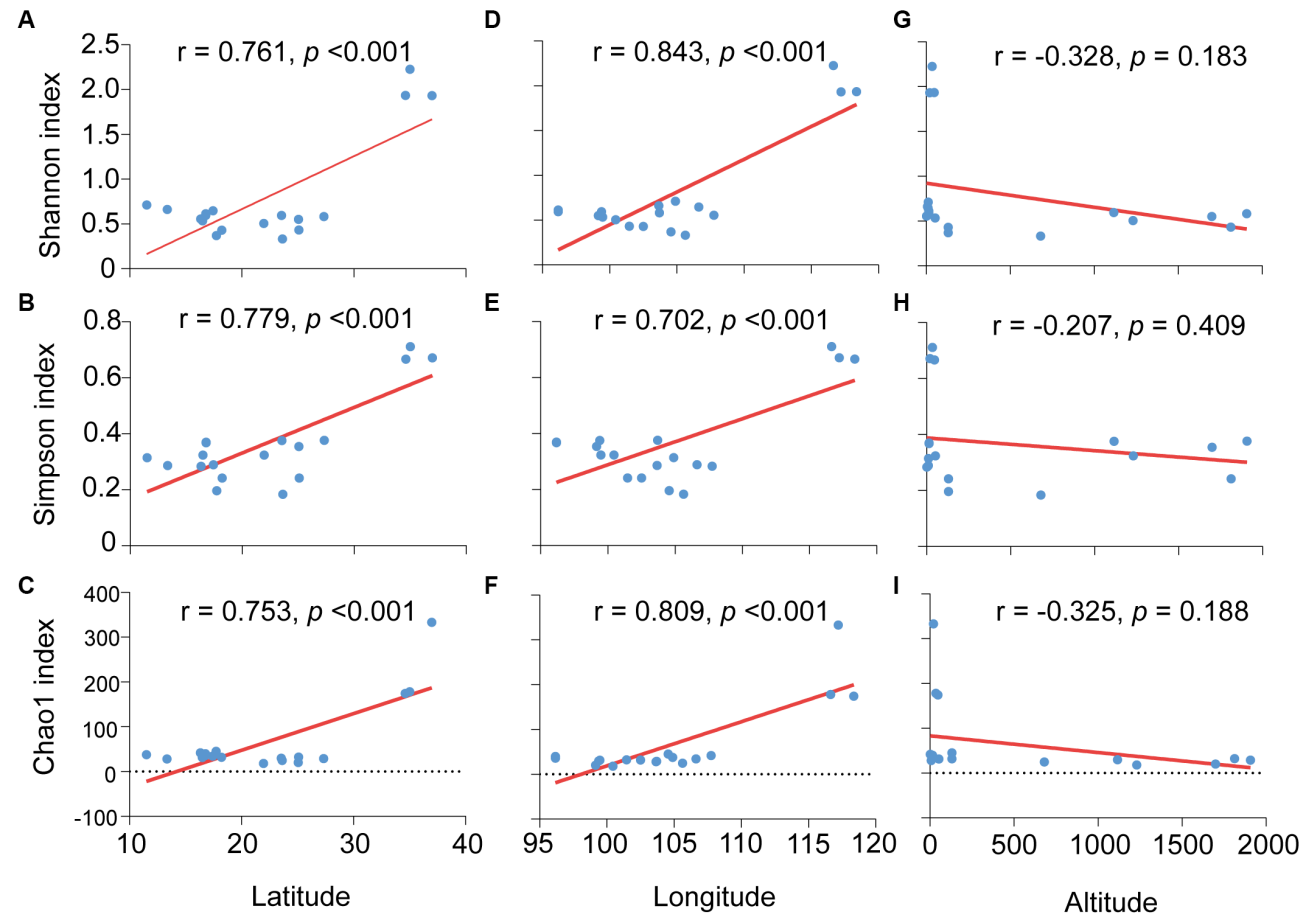
Relationships between the microbiota diverisity indexes and environmental factors in *Sogatella furcifera*. The relationships between latitude and Shannon diversity index **(A)**, Simpson index **(B)** and Chao1 index **(C)**, between longitude and Shannon diversity index **(D)**, Simpson index **(E)** and Chao1 index **(F)**, between altitude and Shannon diversity index **(G)**, Simpson index **(H)** and Chao1 index **(I)** were shown, respectively, all analysis were used by Pearson correlation analysis (SPSS 21.0) based on 2bRAD-M sequencing results. *r*-values and *p* values of each linear regression plots are provided.

**Figure 9:**
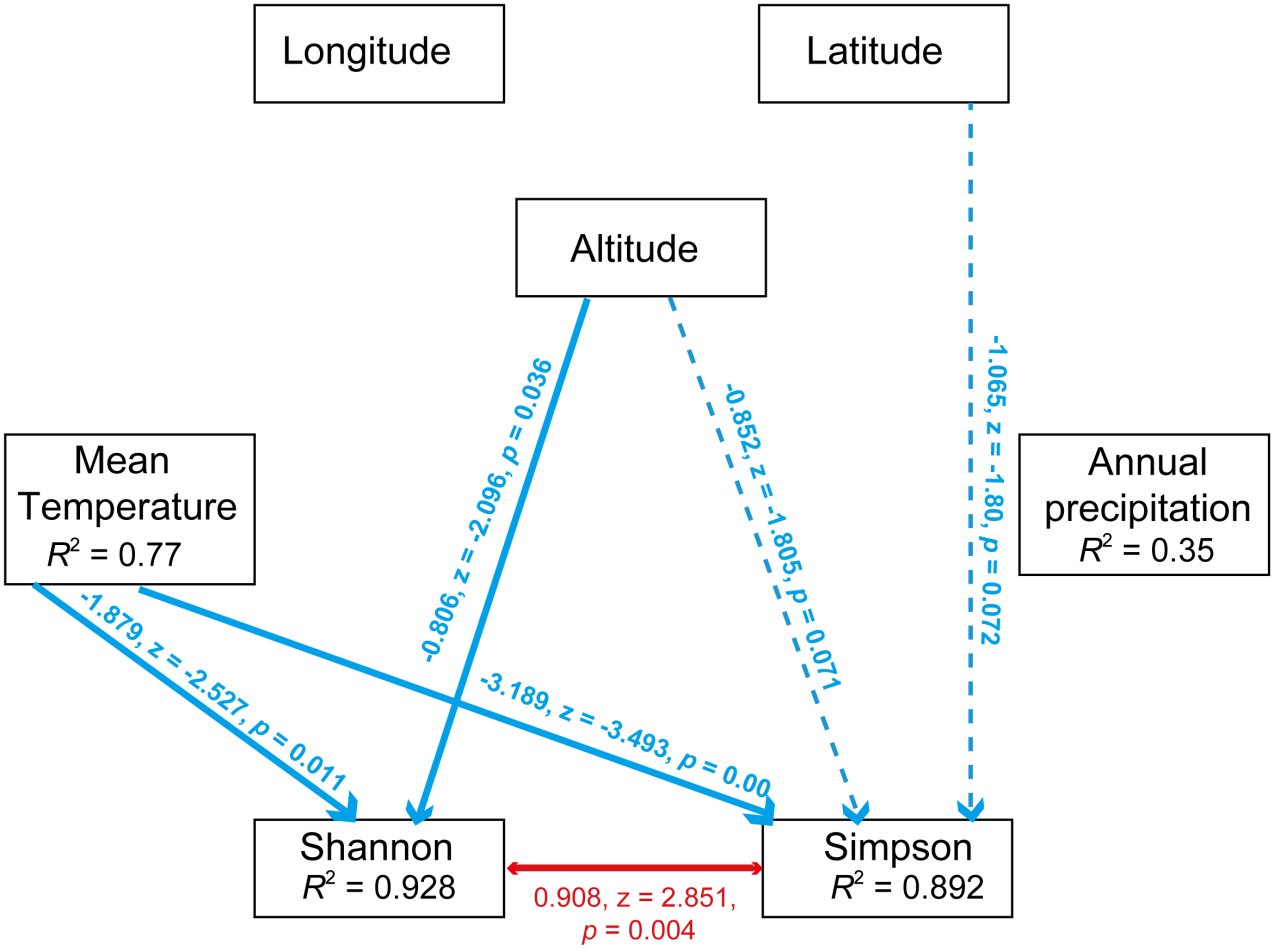
Path diagram for the structural equation model (SEM) for environmental factors (including annual mean temperature, annual precipitation, longitude, altitude and latitude) and diversity indexes (including Shannon index and Simpson index). Statistically significant negative paths are indicated by blue arrows, while positive paths are indicated by red arrows. The r values in each box indicate the amount of variation in that variable explained by the input arrows. Numbers next to arrows are unstandardized slopes.

Furthermore, the effects of environmental factors on microbiota diversity in *S. furcifera* were explored. According to Pearson analysis, both latitude and longitude exerted a positive influence on all three diversity indexes (Shannon, Simpson, and Chao1 indexes) of bacterial communities in *S. furcifera*, whereas altitude had a marginal negative effect on microbiota diversity (Figure 8). In contrast, SEM analysis identified that altitude negatively impacted the Shannon index (*r* = −0.806, z = −2.096, *p* < 0.05, SEM), which differed from the results of Pearson analysis (Figure 8G). SEM analysis uncovered that temperature had a negative influence on both the Shannon index (*r* = −1.879, z = −2.527, *p* < 0.05, SEM) and Simpson index (*r* = −3.189, z = −3.493, *p* < 0.001, SEM) (Figure 9).

## 4 Discussion

Symbiotic bacteria play a critical role in the biology, ecology, and evolution of hosts, and various environmental factors significantly impact bacterial communities in invertebrate hosts. Herein, the effects of symbionts on the microbiota in *S. furcifera* populations and the significant effects of environmental factors on bacterial communities in *S. furcifera* were analyzed. Our findings revealed that the presence of symbionts *Wolbachia* and *Cardinium* in *S. furcifera* negatively influenced the abundance of numerous other bacteria. Additionally, *Wolbachia* infection significantly reduced the diversity of microbial communities in *S. furcifera*. Several environmental factors, including longitude, latitude, temperature, and precipitation, were found to impact the abundance of symbionts and the microbial diversity in *S. furcifera*.

In the current study, the bacterial community composition in *S. furcifera* was abundant, primarily consisting of two dominant symbionts, namely *Wolbachia* and *Cardinium* (Figure 3). These results are consistent with the findings of previous studies on *S. furcifera* microbiota (Li et al., 2020, 2021, 2022). Notably, the presence of a primary symbiont, *Portiera*, in the *S. furcifera* microbiota, with significant infection rates was noted in various populations such as RO (5.03 ± 10.38%, Mean ± SEM) and KO (2.73 ± 1.40%, Mean ± SEM) (Figure 3). The horizontal transmission of symbionts between different arthropod species has been well-documented (Werren et al., 2008; Zhang et al., 2016). Research has established that horizontal transmission can occur between different phloem sap-feeding insect species, accounting for the presence of *Portiera* within *S. furcifera* (Gonella et al., 2015). Previous studies confirmed a vital influence of genetic backgroud on the microbiome in invertebrates (Suzuki et al., 2019; Gupta and Nair, 2020), however, no direct relationship between *S. furcifera* genetic phylogenetic tree and UPGMA hierarchical cluster diagram of microbiome in *S. furcifera* discovered (Supplementary Figures S2, S3).

Interactions among different bacteria, especially symbionts, are complex in arthropods. Specifically, the presence of one symbiont generally influences the infection patterns of other symbionts (Evans and Armstrong, 2006; Zhao et al., 2018; Fromont et al., 2019; Duan et al., 2020). This phenomenon is not surprising, given that symbionts are primarily restricted to bacteriocytes and reproductive tissues within invertebrate hosts, leading to competition for limited nutrition and space (Engel and Moran, 2013; Douglas, 2015; Yang et al., 2019). For example, in *Laodelphax striatellus, Wolbachia* infection negatively affects the abundance of 154 other bacterial genera within hosts (Duan et al., 2020). Conflicting interactions between symbionts, such as *Cardinium* and *Hamiltonella* in whiteflies, have been observed (Zhao et al., 2018), while other bacteria in honeybees decrease the development rate of the primary symbiont *Paenibacillus larvae* (Evans and Armstrong, 2006). Comparable results were obtained in our study; *Wolbachia* negatively influenced the abundance of 13 primary genera of bacteria, while *Cardinium* negatively influenced the abundance of 11 other genera (Figure 4). This observation suggests a competitive relationship among bacteria in *S. furcifera*. However, *Cardinium* infection was positively correlated with the abundance of *Pseudomonas* and *Proteus*, indicating a cooperative interaction between *Cardinium* and these two bacteria (Figure 4B). Similar interactions have been described in other studies. For example, the presence of *Wolbachia* in the fruit fly *Drosophila neotestacea* promotes the infection of *Spiroplasma* (Fromont et al., 2019). In *Laodelphax striatellus, Wolbachia* infection increases the abundance of other bacteria, such as *Spiroplasma* and *Ralstonia* (Duan et al., 2020). These complex interactions among bacteria, especially symbionts, necessitate further exploration.

Symbiont plays a pivotal role in shaping the microbial community of invertebrates and invariably influences the host’s microbiota structure. Symbiont infections frequently lead to a reduction in bacterial diversities in arthropod hosts (Duan et al., 2020; Li et al., 2020, 2022). In the present study, *Wolbachia* infection significantly decreased the diversity of the bacterial community in *S. furcifera* (Figure 5). This finding is in line with the result of previous studies in *Aedes aegypti*, wherein the presence of *Wolbachia* reduced the diversity of resident bacteria in mosquitoes (Audsley et al., 2018). Similar reductions in microbial diversities have been observed in the small brown planthopper *Laodelphax striatellus* (Zhang et al., 2020) and *Drosophila melanogaster* (Ye et al., 2017). These studies collectively underscore the negative influence of symbionts on the bacterial communities of arthropods.

The influence of environmental factors on arthropod microbial communities has been explored in previous research. Studies on global invertebrates indicated that temperature significantly affects the occurrence of *Wolbachia* and *Cardinium* infections in host arthropods (Charlesworth et al., 2019). Similar temperature effects on symbionts were corroborated in other studies (Corbin et al., 2016). However, in this study, the impact of temperature on symbionts (*Cardinium* and *Wolbachia*) was not significant in *S. furcifera* (Figures 6A, 7), suggesting weak temperature effects on symbiont infections in *S. furcifera*. Other environmental factors also influence symbiont infections in hosts. For example, in the spider mite *Tetranychus truncatus*, *Wolbachia* infection was significantly influenced by annual mean temperatures, whereas the rates of *Cardinium* and *Spiroplasma* infections were correlated with altitude (Zhu et al., 2018). Additionally, bacterial communities were significantly affected by geographical distances in stoneflies (Zhu et al., 2022). Herein, the abundance of numerous bacteria in *S. furcifera* were affected by environmental factors, with *Wolbachia* abundance significantly influenced by precipitation and longitude and *Cardinium* abundance not significantly affected by the environment (Figure 6 and Supplementary Figure S3). The differential impact of the environment on *Wolbachia* and *Cardinium* requires further exploration.

In summary, our 2bRAD-M sequencing analysis of 18 *S. furcifera* populations offers a novel and comprehensive perspective of the relationship between bacterial community structure, environmental factors, and symbionts in Asian *S. furcifera* populations. Our observations provide credible evidence that the *Wolbachia* and *Cardinium* symbionts within host whiteflies influence each other and negatively impact the abundance of other bacteria. Importantly, *Wolbachia’s* presence negatively affected microbial diversity, with both symbiont and microbiota diversities being significantly influenced by various environmental factors. However, further studies are necessary to elucidate the mechanisms by which environmental factors influence the diversity of bacterial communities in different *S. furcifera* populations across global ecological timescales.

## Data availability statement

The original contributions presented in the study are publicly available. This data can be found here: NCBI, PRJNA1055281.

## Ethics statement

The manuscript presents research on animals that do not require ethical approval for their study.

## Author contributions

KY: Conceptualization, Data curation, Funding acquisition, Investigation, Methodology, Writing – original draft, Writing – review & editing. H-YZ: Investigation, Methodology, Validation, Writing – review & editing. PW: Supervision, Writing – original draft. G-XJ: Formal analysis, Validation, Conceptualization, Writing – original draft, Writing – review & editing. DC: Conceptualization, Formal analysis, Funding acquisition, Supervision, Validation, Writing – original draft, Writing – review & editing.

## Footnotes

^1^ https://worldclim.org/
