# Supplementary material for: Both symbionts and environmental factors contribute to shape the microbiota in a pest insect, Sogatella furcifera
Source: Front Microbiol. 2024 Jan 24;14:1336345. doi: 10.3389/fmicb.2023.1336345 (PMC10860895; doi:10.3389/fmicb.2023.1336345)
Supplement: Supplementary file 1 [file Data_Sheet_1.docx]

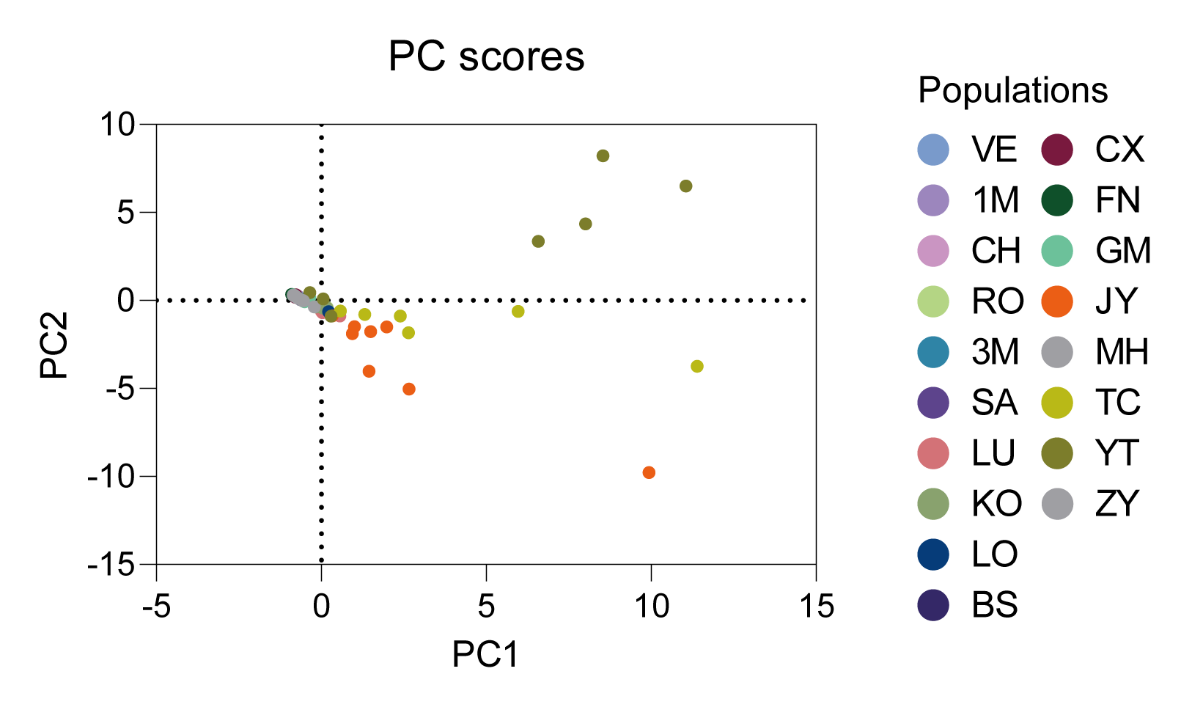


**Fig. S1.** PCoA of bacterial communities all 18 *Sogatella furcifera* populations based on Bray-Curtis similarity analysis.


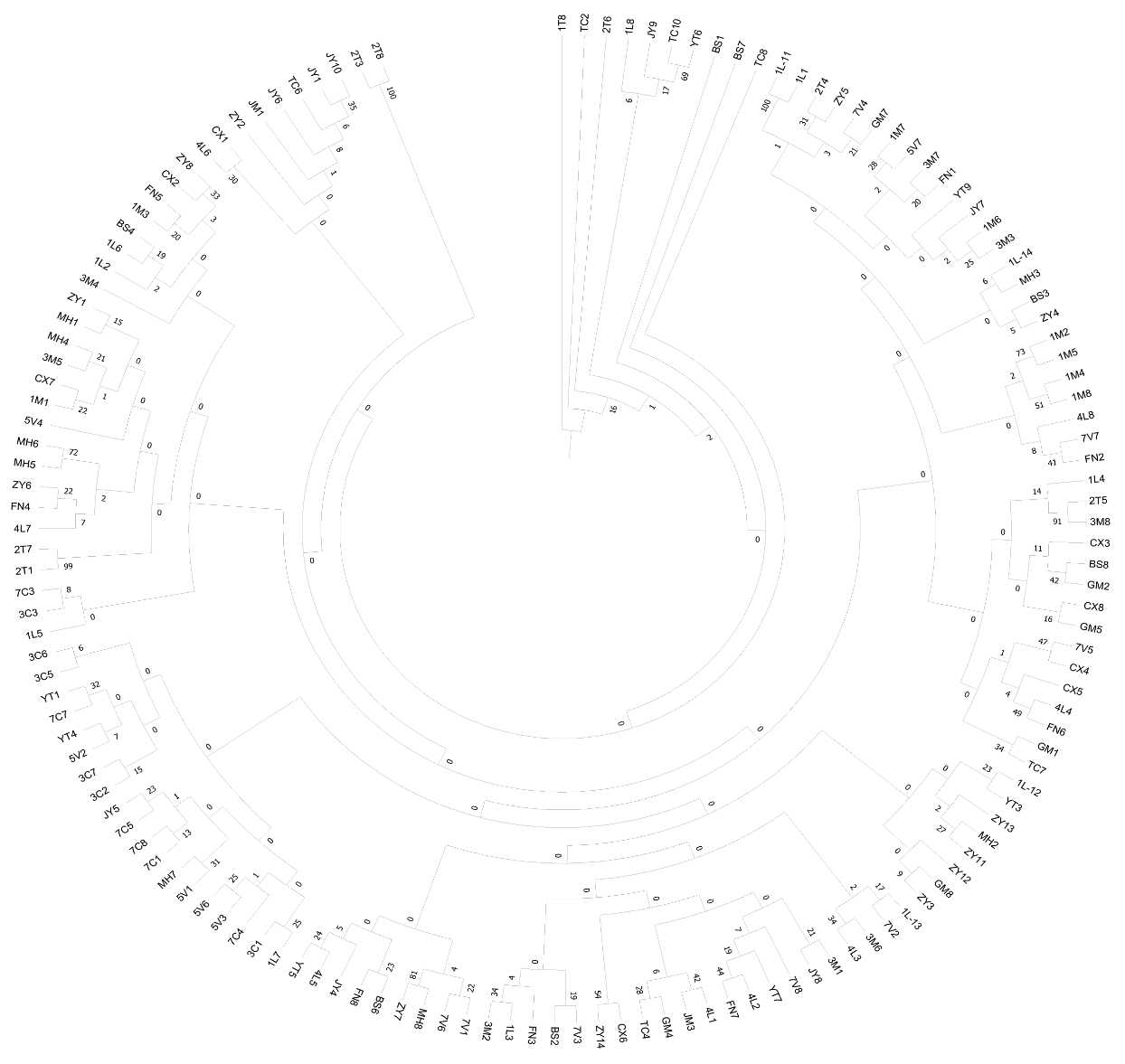


Fig. S2. Phylogenetic tree of different *Sogatella furcifera* populations with 2bRAD sequencing results.


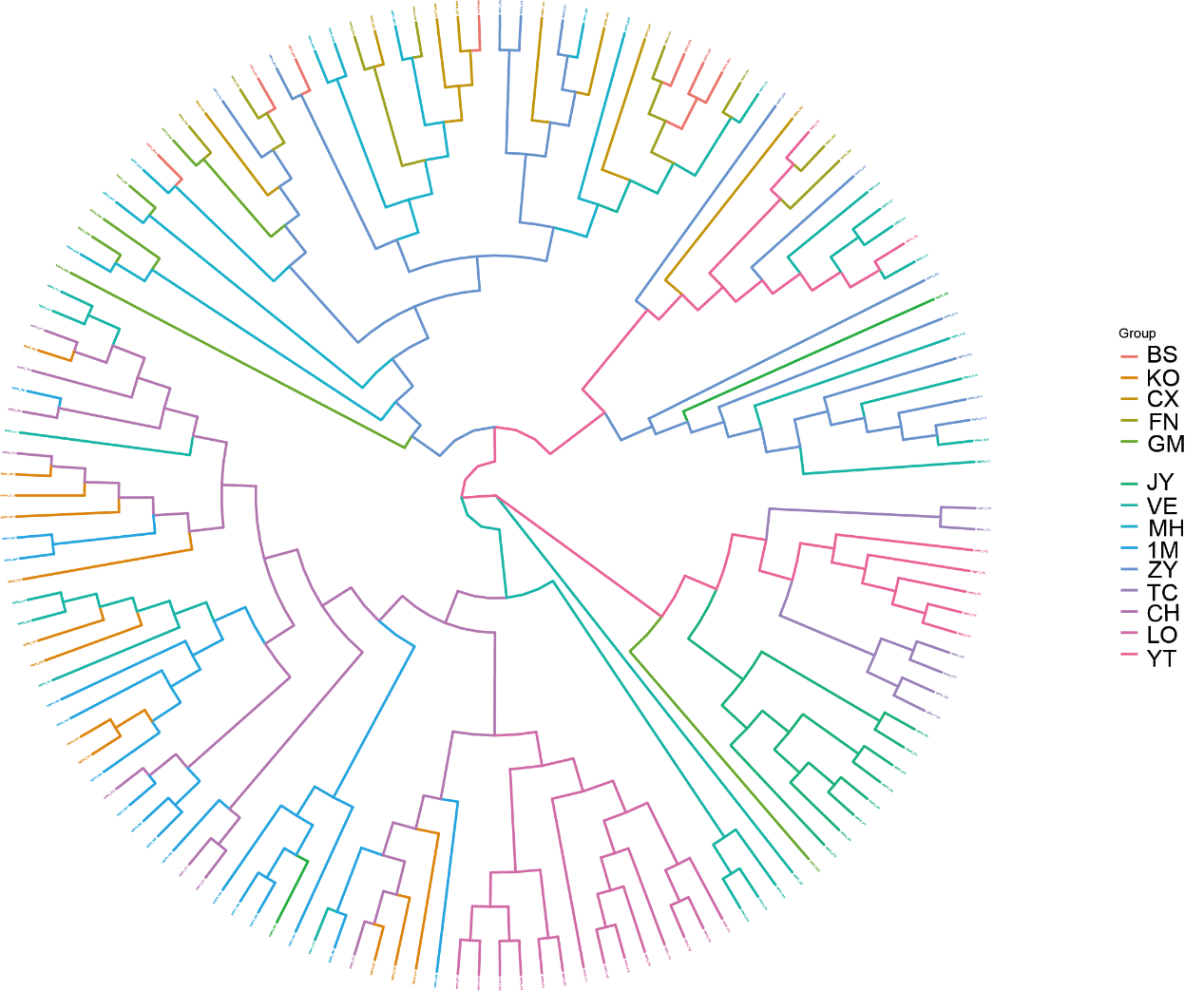


**Fig. S3.** UPGMA hierarchical cluster diagram of different *Sogatella furcifera* populations with 2bRAD-M sequencing results.


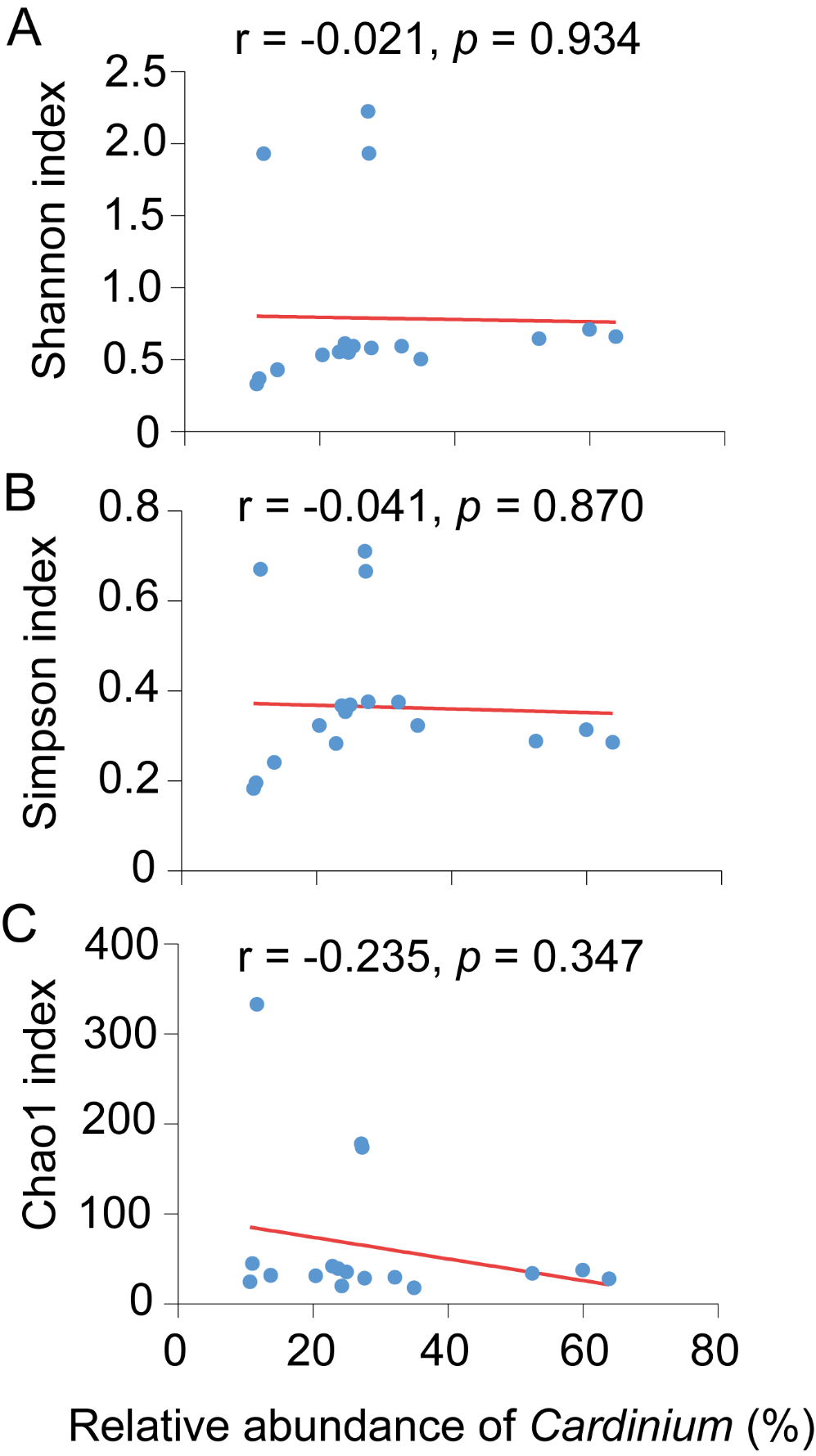


**Fig. S4.** Relationships between the abundance of Cardinium and the Shannon diversity index (A), Simpson index (B) and Chao1 index (C) among all 18 *Sogatella furcifera* populations by Pearson correlation analysis (SPSS 21.0) based on 2bRAD-M sequencing results. *r*-values and *P* values of each linear regression plots are provided.


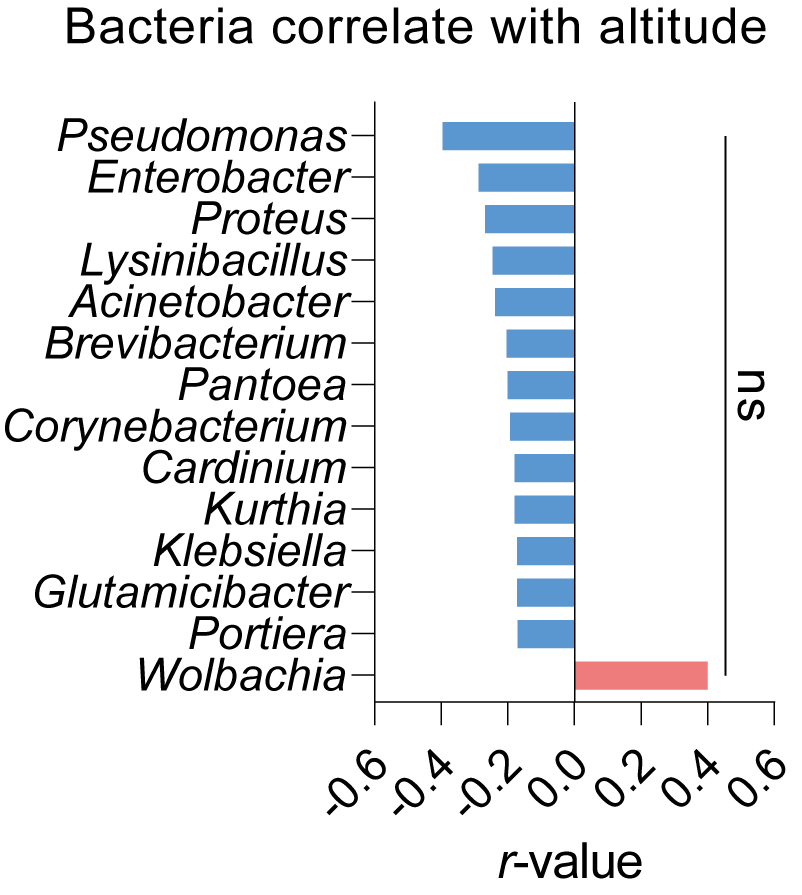


**Fig. S5.** Relationships between the proportions of main 14 bacteria and the altitude among all 18 *Sogatella furcifera* populations by Pearson correlation analysis (SPSS 21.0) based on 2bRAD-M sequencing results. *r*-values and *P* values of each linear regression plots are provided. “ns” means no significant; asterisks indicate significant difference the two compared group, *, P < 0.05; **, P < 0.01; ***, P < 0.001.
